# The 115 Years Old Multicomponent Bargellini Reaction: Perspectives and New Applications

**DOI:** 10.3390/molecules26030558

**Published:** 2021-01-21

**Authors:** Marta Serafini, Ilaria Murgia, Mariateresa Giustiniano, Tracey Pirali, Gian Cesare Tron

**Affiliations:** 1 Dipartimento di Scienza del Farmaco, Università del Piemonte Orientale, Largo Donegani 2, 28100 Novara, Italy; marta.serafini@uniupo.it (M.S.); ila.murgia@hotmail.it (I.M.); 2 Dipartimento di Farmacia, Università degli Studi di Napoli “Federico II”, Via D. Montesano 49, 80131 Naples, Italy

**Keywords:** Bargellini reaction, multicomponent reaction, chloroform, chlorobutanol, α-aryl isobutyric acids

## Abstract

Despite its uniqueness, the Bargellini multicomponent reaction remains barely known by the most part of chemists. This can be ascribed to the fact that this transformation has not been adequately reviewed in the classic books of named reactions in organic chemistry. Nevertheless, several works on this reaction have been carried out over the years, many of them were written in Italian in the period 1929–1966. In this review article we extensively cover, in a chronological order, the most important applications of the Bargellini reaction reported to date, with the hope that this knowledge-sharing will help chemists to properly use this multicomponent transformation and imagine novel reactivities based on it.

## 1. Introduction

### 1.1. The Discovery of a Novel Reaction

**Scheme 1:**
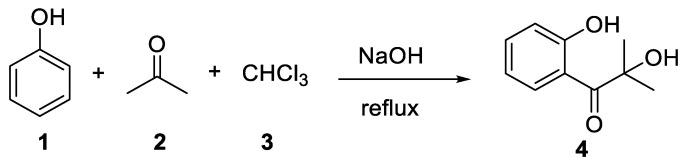
The reaction reported by Link in 1894.

The story of this reaction begins with a German patent published by Link in 1894 [1] in which the 2-hydroxy-1-(2-hydroxyphenyl)-2-methylpropan-1-one (**4**) was prepared by heating a mixture of phenol (**1**), acetone (**2**) and chloroform (**3**) for 5–6 h (Scheme 1).

**Scheme 2:**
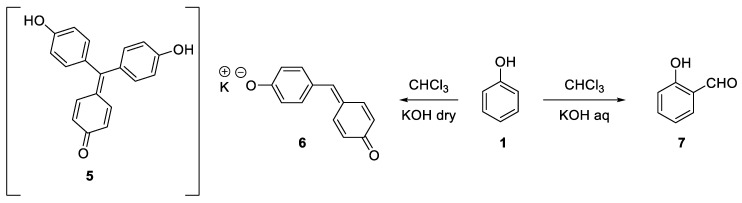
Guareschi-Lustgarden vs. Reimer-Tiemann reaction.

At that time, the reaction between phenols, chloroform and solid sodium hydroxide was already well known. Indeed, it had been reported for the first time in 1872 by Icilio Guareschi, using the pre-formed sodium phenate [2], and later by Sigmund Lustargarden [3]. This reaction was used as a colorimetric method to detect less than 0.1 mg of phenol thanks to the supposed formation of the strongly red-purple colored rosolic acid derivative (**5**). Actually, the true composition of this precipitate is not known and it could also be the sodium salt of the quinone methide **6** [4,5]. This reaction was later investigated by Reimer and Tiemann who discovered that under aqueous conditions *o*-formylphenols (**7**) were obtained (Scheme 2) [6,7].

Although not reported in the original patent, as an archeochemist, it is possible to imagine that acetone was used as an inert solvent by Link, but, contrary to expectations, it reacted. The parallelism with the discovery of the Passerini multicomponent reaction is stunning [8].

**Scheme 3:**
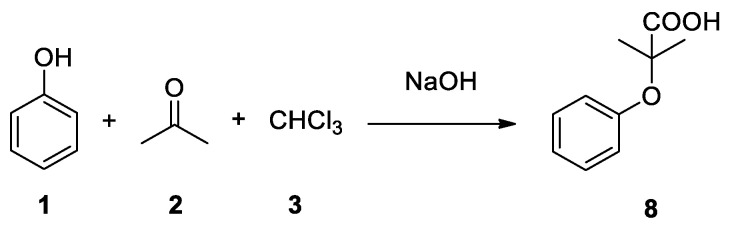
The reaction revised by Bargellini in 1906.

However, structure **4** proved to be wrong and 27-year old Guido Bargellini in 1906 [9,10] showed that the real product of the reaction was the α-phenoxyisobutyric acid **8** (Scheme 3). Bargellini demonstrated that no phenolic hydroxyl was present as the molecule was neither acetylated nor gave the methyl ether. Moreover, no reactions with hydroxylamine or phenylhydrazine occurred, confirming the absence of the keto group. The compound was soluble in a carbonate solution and it could be re-precipitated by addition of HCl, indicating the presence of a carboxylic acid. 

The same compound **8** had already been obtained in 1900 by Bischoff [11] via basic hydrolysis of the corresponding ethyl ester prepared by reaction between sodium phenate and ethyl 2-bromo-2-methylpropanoate. 

**Scheme 4:**
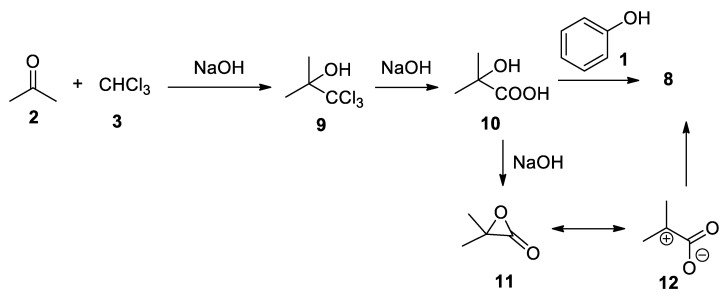
Sequential reaction mechanism proposed by Bargellini.

From a synthetic point of view, the Bargellini reaction allows for the direct synthesis of aryl ether-bearing sterically hindered carboxylic acids. In the same paper, Bargellini demonstrated the generality of this reaction that works also with other phenolic components, such as α-naphthol, β-naphthol, *o*- and *p*-cresol, and thymol. Regarding the mechanism of the reaction, Bargellini proposed the formation of acetonechloroform **9**, followed by its hydrolysis to 2-hydroxy-2-methylpropanoic acid **10** and final condensation with phenol **1** (Scheme 4). As we will see later, this hypothesis proved to be wrong, in spite of the fact that it was demonstrated by Bargellini that heating for 6 h in a bain-marie compounds **10** and **1**, in the presence of sodium hydroxide, **8** could be obtained in high yield [9]. 

In order to explain the reactivity of 2-hydroxy-2-methylpropanoic acid (**10**), it is possible to consider that **10** under strong basic condition leads to the formation of α-lactone **11**. α-Lactones are unstable and reactive intermediates, being attacked by nucleophiles on the α carbon instead of the carbonyl group. This apparently strange behavior can be rationalized considering that α-lactones can be represented by two resonance forms of equal energy, that is, the closed form **11** and an open 1,3-dipolar form **12**. High temperatures and polar solvents facilitate the formation of **12** [12,13]. 

### 1.2. Guido Bargellini 

Guido Bargellini was born in 1879 in Tuscany, more precisely in Roccastrada, a small village near Grosseto, and he took his degree in Chemistry in Rome in 1902. For two years he was laboratory assistant at the University of Siena, and, after a stint in the laboratory of Emil Fisher, he came back to the University of Rome as an assistant, first to Stanislao Cannizaro, and later to Emanuele Paternò. From 1921–1924 he was Professor at the University of Sassari, then of Siena and finally of Rome, where he remained until his death in 1963 [14]. His research activity was mainly based on the chemistry of natural substances like santonin, chalcones, flavones, flavanols and phenylcoumarins. At least three eponymous reactions were discovered by Bargellini: (i) the multicomponent reaction described in this review; (ii) a chromatic reaction of polyoxyflavones with sodium amalgam known as Bargellini’s test [15], and (iii) the synthesis of phenylcoumarins by the reaction between sodium phenylacetate and *o*-hydroxyacetophenones in the presence of acetic anhydride known as Bargellini’s condensation [16,17]. 

**Figure 1:**
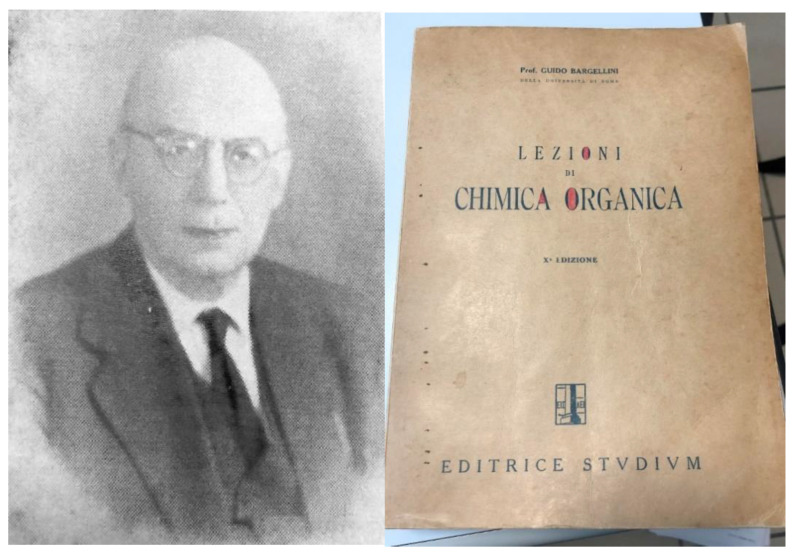
Professor Bargellini and the legendary organic chemistry textbook he authored (this copy belongs to one of the authors (GCT)).

Next to his research activity, he devoted most of his time to the academic teaching, in particular of organic chemistry and war-chemistry (*Chimica di Guerra*). His book “*Lezioni di chimica organica*” (Organic chemistry lessons) was one of the first organic chemistry textbooks written by an Italian author. The book had a vast diffusion, reaching the XIth edition and being the leading text for a generation of Italian chemists (Figure 1). 

## 2. The Bargellini Reaction

From now on, we will name Bargellini reactions those transformations in which ketones react with chloroform and then with a nucleophile. Conversely, we will not include in this review those reactions where trichloromethyl carbinols, obtained reacting an aldehyde with chloroform, are used. Indeed, these transformations can be ascribed to other two named reactions: the Jocic-Reeve [18] and the Corey-Link reactions [19]. These transformations have been covered by other recent reviews [20]. In this review, we report, in a chronological order, the most important applications of the Bargellini reaction reported so far, while in the conclusions we provide a practical guide to properly use and essay this multicomponent transformation, together with a perspective on possible unprecedented applications. 

### Reaction Mechanism and Formation of By-Products

**Scheme 5:**
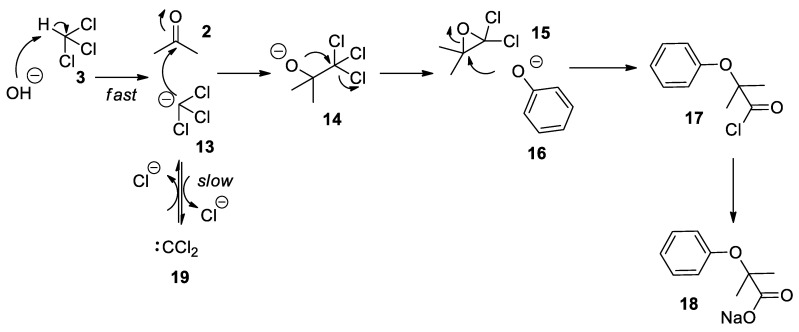
The most widely accepted mechanism for the Bargellini reaction.

The revised and nowadays accepted mechanism of the Bargellini reaction is due to the work of Weizmann et al. [21]. As already proposed by his inventor, the first step is the formation of **14** which undergoes a fast intramolecular substitution reaction to form the 2,2-dichloro-3,3-dimethyloxirane **15**, referred to as the Bargellini epoxide. This epoxide can then undergo a nucleophilic attack by phenolate **16** in an S_N_2 reaction with substantial S_N_1 character to form an acyl chloride intermediate **17** which is next hydrolyzed to carboxylate **18** due to the strong basic conditions of the medium (Scheme 5). Seminal contributions by Hine who carried out kinetics experiments on basic hydrolysis of chloroform showed that proton abstraction on chloroform (**3**) to form the trichloromethane anion **13** is a fast process, while the α-elimination to dichlorocarbene **19** is a slower event which sees the dichlorocarbene in equilibrium with the trichloromethylide anion (Scheme 5) [22,23]. 

The use of bromoform is possible and it has been reported [24], but chemists should be aware that: (i) dibromocarbene is much more reactive than dichlorocarbene, which alters the equilibrium between these two species, (ii) the basic hydrolysis of CHBr_3_ is six times faster compared to CHCl_3_. 

The base-induced formation of haloform carbanion follows the order I > Br > Cl > F, while the relative ease to lose a halogen from the trihalocarbanion to give a dihalocarbene is Br > I > Cl > F. Finally, the reactivity of dihalocarbenes follows the following order: F > Cl > Br > I. 

After extensive investigations, it is now established that the typical Bargellini conditions require one equiv. of phenol, three equiv. of acetone, four equiv. of chloroform and six equiv. of freshly pulverized sodium hydroxide in THF. Moreover, a careful control of the temperature is mandatory, being the reaction highly exothermic.

**Scheme 6:**
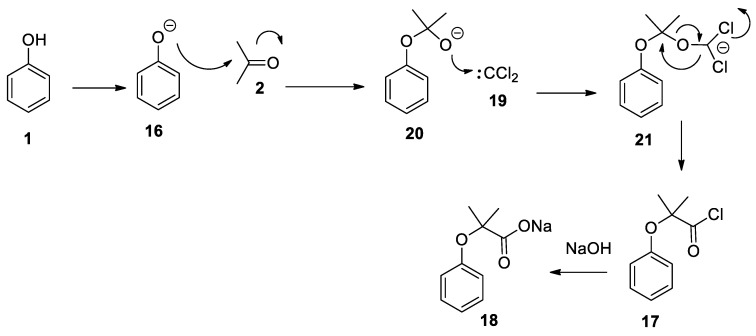
An alternative, but highly improbable, proposed mechanism of reaction.

Another possible mechanism for the Bargellini reaction, when performed under strict anhydrous conditions, has been proposed and it is based on the reaction of phenolate **16** with acetone **2**. The obtained intermediate **20** can react with dichlorocarbene **19**, generated in situ by the α-decomposition of the trichloroformate anion, to give **21**. An intramolecular rearrangement of **21** gives the corresponding acyl chloride **17** which is immediately hydrolyzed to carboxylate **18** (Scheme 6). To prove the proposed mechanism, the authors reacted phenol with acetone under basic conditions isolating the intermediate 2-phenoxypropan-2-ol [25]. When the latter compound was reacted with chloroform under anhydrous basic conditions, the Bargellini adduct was formed. The authors mention that the presence of water prevents the formation of the product, as dichlorocarbene rapidly reacts with it to form formic acid. Although intriguing, the intramolecular rearrangement of **21** appears unlikely to the authors of this review as the hemiacetal intermediate 2-phenoxypropan-2-ol under basic conditions can re-form acetone and phenolate.

It is important to note that the formation of the trichloromethane anion and its attack to acetone to form the oxirane is known to be a very fast reaction, occurring even at low temperature [26], while the formation of dichlorocarbene requires higher temperature.

**Scheme 7:**
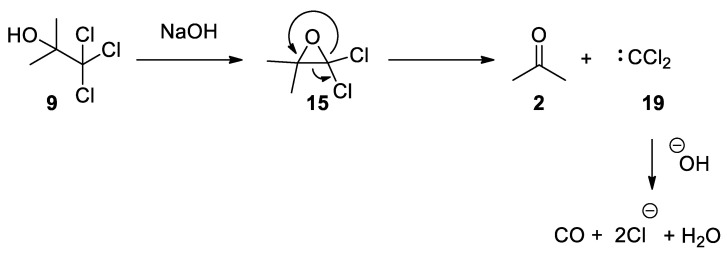
Commercially available chlorbutanol can be used to form Bargellini epoxide.

The Bargellini epoxide **15** can also be formed using the commercially available chlorobutanol **9** (also known as chloretone or acetonechloroform), which has been used in medicine as sedative-hypnotic and bacteriostatic. Treatment of chlorobutanol in the presence of sodium hydroxide generates the *gem*-dichloroepoxide **15**, which can be directly intercepted by nucleophiles. From kinetic data, it has been suggested that Bargellini epoxide might revert to give acetone and dichlorocarbene, which is then hydrolyzed to form carbon monoxide [27,28] (Scheme 7).

**Scheme 8:**
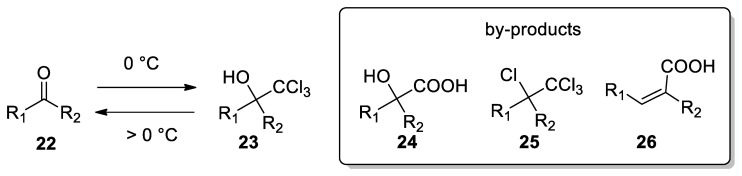
Careful control of temperature plays a pivotal role in the stability of trichloromethyl carbinol anions and formation of by-products.

Several procedures for the formation of other trichloromethyl carbinols **23** emerged from the literature. For example, aldehydes and ketones can be converted in trichloromethyl carbinols by reacting chloroform, 5% TEBAC and 50% sodium hydroxide. A very fast addition of the base at 0 °C can give higher yields, up to 80% [26], while at reaction temperature highers than 0 °C, the yield is reduced, due to reversion of carbinol **23** to the starting materials **22** and chloroform. Possible by-products deriving from this transformation with ketones are α-hydroxyacids **24**, α-chloro acids **25** and α,β-unsaturated acids **26** [29] (Scheme 8). 

**Scheme 9:**
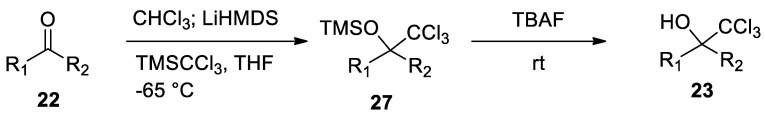
A novel synthesis of 2,2,2-trichloromethyl carbinols.

In order to avoid the formation of the above-mentioned by-products, 2,2,2-trichloromethyl carbinols **23** have been recently prepared by TBAF-mediated silyl deprotection of trimethyl(trichloromethyl)silane TMSCCl_3_ previously obtained reacting the ketone with chloroform and LiHMDS. This procedure avoids the use of strong basic conditions [30] (Scheme 9). 

Generation of the trichloromethane carbanion under neutral conditions can be also achieved heating at reflux sodium trichloroacetate, which loses carbon dioxide. The carbanion decomposes to dichlorocarbene if there are no acidic protons in the medium [31]. 

**Figure 2:**
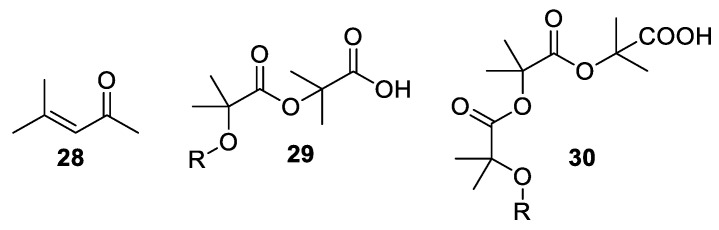
By-products deriving from the Bargellini reaction.

Over the years, other by-products, which can reduce the yield and make the isolation of the desired product difficult, have been isolated from the Bargellini reaction. The awareness of these side-reactions is important in order to minimize their occurrence. A very well-known by-product is for example mesityl oxide **28**, which emerges from the self-condensation of acetone. Other reported by-products are those that form from the α-aryloxyisobutyric acid which reacts further with one or two Bargellini epoxides, producing compounds **29** and **30** (Figure 2).

It has been suggested that the nature of the counterion can affect the degree of formation of these by-products. Potassium and tetraalkylammonium carboxylate salts of Bargellini products appear to be more reactive towards Bargellini epoxides, while sodium carboxylate is less prone to react further. LiOH and DBU act similarly to KOH, while *t*-BuONa leads to an extensive decomposition. In order to avoid the formation of mesityl oxide by-product, the best choice is a slow addition of chloretone solution in acetone to the reaction mixture containing pulverized NaOH, acetone and phenol, under cooling conditions in order to maintain the temperature between 25–30 °C. This represents the best mode of operation in order to maintain high yields and reduce the formation of by-products [31,32].

Recently, dimeric by-products **29** and **30** have been reported as main products with yields ranging from 92 to 41% when refluxing an excess of chloroform, sodium hydroxide and dry acetone for 40–53 h [33].

**Scheme 10:**
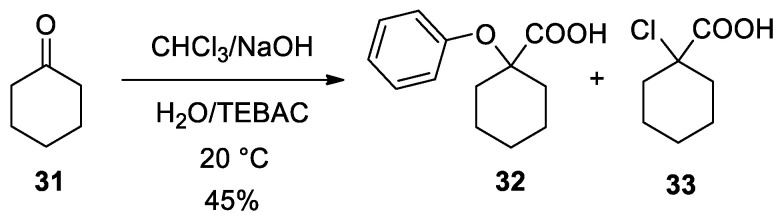
A by-product (**32**) which can be obtained from the Bargellini reaction with cyclohexanone when poor nucleophiles are present.

Use of different carbonyl compounds other than acetone can cause the formation of other specific by-products. For example, with cyclohexanone (**31**), the Bargellini product **32** was formed together with the 1-chlorocyclohexane-1-carboxylic acid (**33**). The formation of this by-product is to ascribe to the use of less reactive trapping nucleophiles. (Scheme 10).

**Scheme 11:**
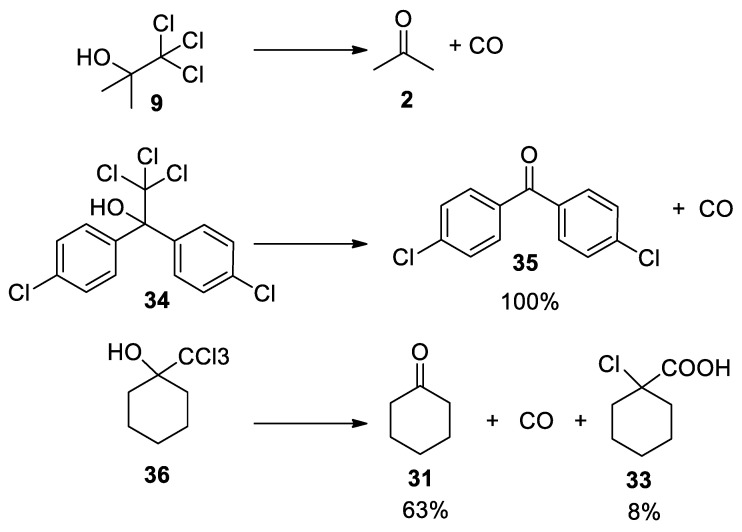
Decomposition of 2,2,2-trichloromethylcarbinols under basic conditions.

As already discussed above, the trichloromethyl carbinols can decompose if not intercepted by an external nucleophile. Experiments in which different trichloromethyl carbinols reacted in the presence of 10% KOH at 0 °C reveal the formation of the corresponding ketones and carbon monoxide. Again, we note that the source of carbon monoxide is not the trichloromethane anion, but the dichloroepoxide, which slowly breaks down into the carbonyl component and dichlorocarbene that is next hydrolyzed to carbon monoxide [18] (Scheme 7 and Scheme 11).

## 3. Applications of Bargellini Reactions

### 3.1. Reaction with Phenols

After the seminal publication of Bargellini, it was only in 1947 that Galimberti and De Franceschi reported the Bargellini reaction with other phenols [34]. Later, additional works reported the synthesis of dimethylaryloxyacetic acids obtained with other substituted phenols [35,36].

In general, acetone, cyclohexanone, cyclopentanone and methyethylketone are good carbonyl partners in the Bargellini reaction, while benzophenone and acetophenone are not reactive enough. 

Clofibric acid is a well-known drug used to reduce the level of triglycerides in the blood. Even if it can be synthesized starting from *p*-chlorophenol and 2-bromo-2-methylpropanoic acid, [37,38] as an alternative the Bargellini reaction can be used, heating the reagent at reflux for 4 h to yield the desired compound in 37% yield [32,39]. Note that the SN_2_ reaction between phenols and the sterically demanding 2-bromo-2-methylpropanoic acid is not effective with *ortho*-substituted phenols, leading to yields ranging from 15 to 60% after prolonged heating under strong basic conditions (even 7 days!). In this case, the Bargellini reaction, which does not suffer from steric hindrance, works much better and should be considered as the first choice [32]. 

The FT-IT technique was used to monitor the reaction mixture using chlorobutanol and sodium hydroxide. A short-lived species (<5 min) with a unique peak at 758 cm^−1^ was observed. It has been suggested that this short-lived intermediate is the dimethyl dichloroepoxide [32]. 

### 3.2. Reaction with Other Nucleophiles

After the use of phenol and its derivatives, other chemists sporadically reported the successful exploitation of the Bargellini reaction using different types of nucleophiles. Herein, we report the nucleophiles used so far in a chronological order.

In 1929, while studying the Hofmann reaction for the formation of aromatic isocyanides, Gastone Banti from the University of Florence reported the reaction between aniline and chlorobutanol in the presence of sodium hydroxide to afford 2-methyl-2-(phenylamino)propanoic acid. The same conditions were also used for the *o*-phenyldiamine to provide quinazoline [40]. Unfortunately, this strategy was not studied in detail by the author and this transformation would be re-discovered later, with better reaction conditions and higher yields as described below. 

**Figure 3:**
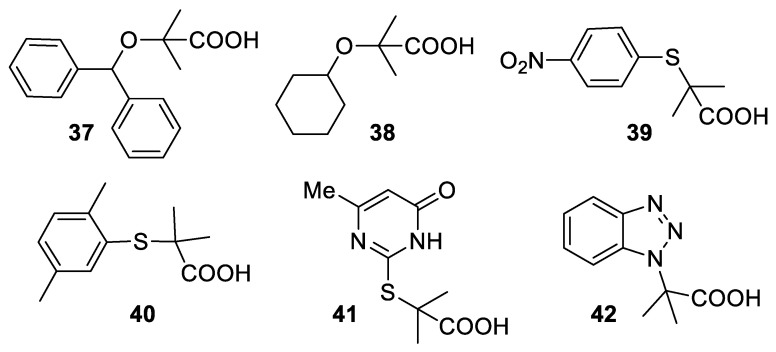
Compounds obtained with the Bargellini reaction.

In 1947, besides the reaction with phenols, Galimberti and De Franceschi, in the same publication reported the formation of Bargellini adducts using alcohols (i.e., benzydrol, cyclohexanol), thiols (i.e., *p*-nitrothiophenol, thio-*p*-xilenol), 4-methylthiouracil, and benzotriazole [34] (Figure 3). The authors stated that aryl mercaptans reacted better than phenols, but unfortunately no yields were reported in the publication. In another report, thiophenol, *p*-methoxythiophenol, *p*-nitrothiophenol and *p*-methylthiophenol were reacted under Bargellini conditions, yielding the desired α-arylthioisobutyric acids in yields between 72 and 52% [41,42].

**Scheme 12:**
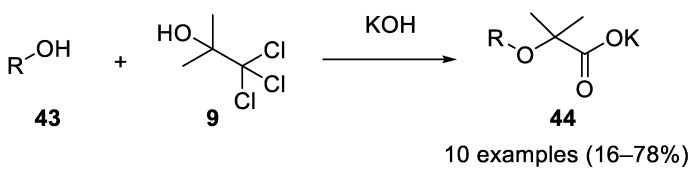
Aliphatic alcohols as nucleophilic partners in the Bargellini reaction.

Using acetonechloroform (**9**), four equiv. of potassium or sodium hydroxide and an aliphatic alcohol **43** at 0 °C, the corresponding Bargellini adducts **44** were obtained in 1948. The reaction worked with primary, secondary and tertiary alcohols, with the primary ones being more reactive. α-alkoxyisobutyric acids can also be dealkoxylated to form metacrylic acids [16] (Scheme 12).

**Figure 4:**
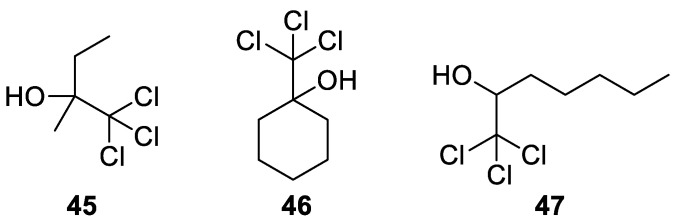
Trichloromethyl carbinols used in the Bargellini reaction with alcohols.

Besides acetonechloroform (**9**), other chlorinated alcohols **45**–**47** have been used, such as those shown in Figure 4.

**Scheme 13:**
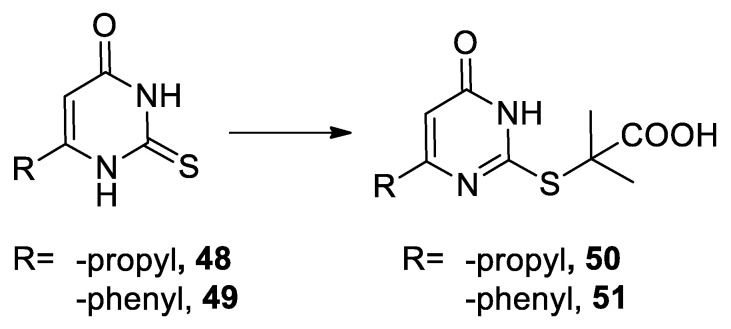
Reaction with thiouracils.

In 1963 thiouracil derivatives **48** and **49** reacted under Bargellini conditions [43] (Scheme 13).

**Scheme 14:**
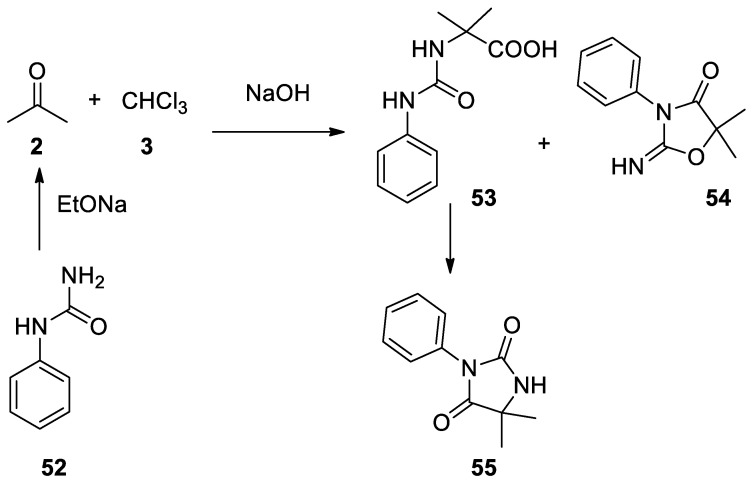
Reaction with phenylurea.

In 1963 phenyl urea (**52**) reacted under Bargellini conditions to give two products: an open-chain product **53**, which under heating can cyclize affording 3-phenyl-5,5-dimethylhydanthoin **55**, and a cyclic product **54** [44] (Scheme 14). 

**Scheme 15:**
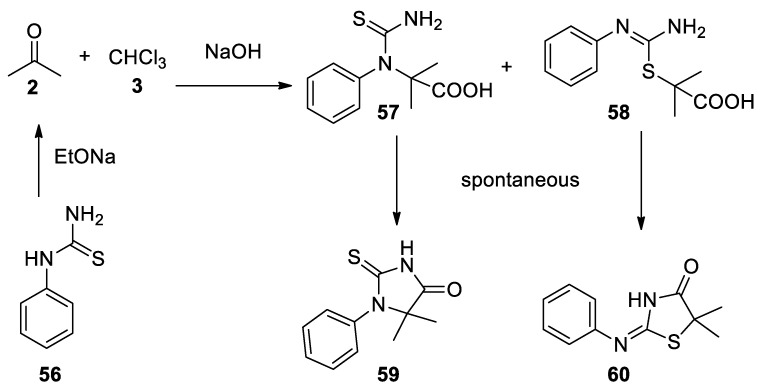
Reaction with phenylthiourea.

In the same year the reaction with phenylthiourea (**56**) was reported. In this case, the two open-chain Bargellini adducts **57**–**58** were not stable and spontaneously dehydrate to form cyclic compounds **59**–**60** [45] (Scheme 15).

**Scheme 16:**
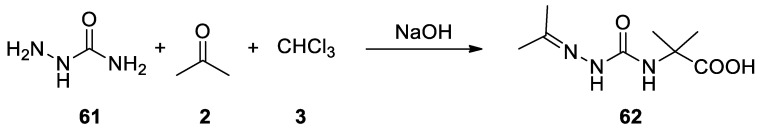
Reaction with semicarbazide.

In 1964 semicarbazide (**61**) and thiosemicarbazides reacted in the Bargellini reaction to give 2-methyl-2-(2-(propan-2-ylidene)hydrazine-1-carboxamido)propanoic acid (**62**), and the corresponding thio derivative [46] (Scheme 16). 

**Figure 5:**
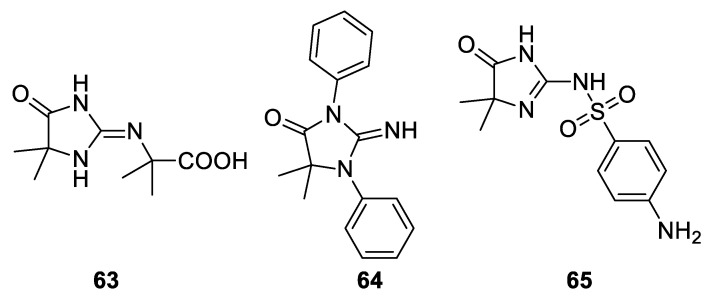
Products obtained from the reaction with guanidine, diphenylguanidine and sulfaguanidine.

A less trivial result was obtained in 1966 reacting guanidine under Bargellini conditions: according to the available spectroscopic methods, the authors stated that compound **63** was obtained. Also, diphenylguanidine and sulfaganidine underwent the Bargellini reaction giving compounds **64** and **65**, respectively [47] (Figure 5).

**Scheme 17:**
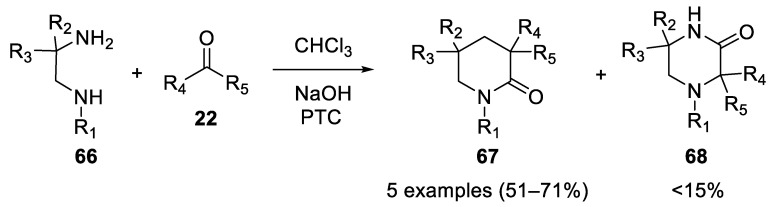
Reaction with *N*-substituted-2-methyl-1,2-propanediamines.

In 1980 *N*-substituted-2-methyl-1,2-propanediamines **66** reacted in the Bargellini reaction in the presence of 50% aqueous sodium hydroxide, chloroform and acetone under phase-transfer catalysis to afford 1,3,3,5,5-pentasubstituted-2-piperazinones **67** [48] (Scheme 17).

**Scheme 18:**
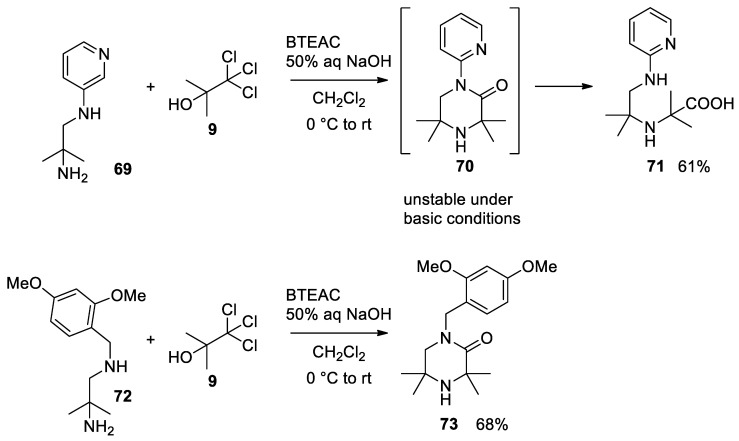
Bargellini reaction in the presence of a second nucleophilic group.

The reaction was carried out also using chlorobutanol, giving improved yields. This strategy shows that when a second nucleophilic group is present (as in **69**), the ephemeral acyl chloride can be intramolecularly intercepted by the second nucleophile, before being hydrolyzed to carboxylic acid **71**. It is possible that the lactam undergoes basic hydrolysis forming the open chain product. On the other hand, it has been shown that electron donating groups on the secondary amine (i.e., **72**) are beneficial to the Bargellini reaction, giving a lactam resistant to basic hydrolysis (compound **73**, Scheme 18) [49]. 

**Scheme 19:**
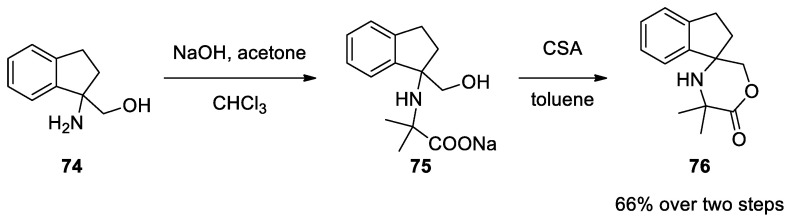
Reaction with 1,2-diaminoalchols.

1,2-Diaminoalchols **74** can also react under classical Bargellini conditions. Dehydration with CSA in toluene forms morpholinone **75**. Direct formation of morpholinone, even under phase transfer conditions (PTC), is not possible as the lactone moiety undergoes hydrolysis due to the basic reaction medium that favors the open form **75** [50,51,52] (Scheme 19).

**Scheme 20:**
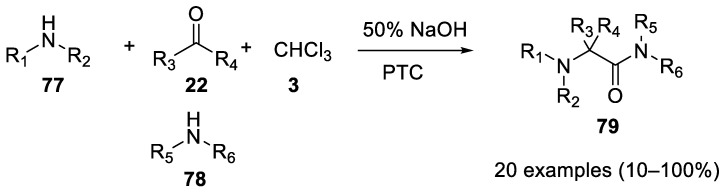
Reaction with aliphatic amines.

In 1980 aliphatic amines **77** were shown to be good partners in Bargellini reactions under PTC conditions. In this case, using an excess of the amine, the intermediate acyl chloride is not hydrolyzed to a carboxylic acid, but rather it reacts with the secondary amines (identical or different from the first one). The net result of this reaction is the formation of hindered acyclic α-aminoacetamides **79**. With carbonyl compounds other than acetone, imines are predominantly formed [53] (Scheme 20). 

**Scheme 21:**
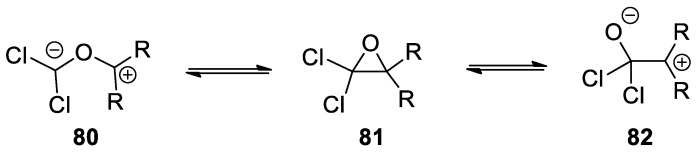
Resonance forms of the Bargellini epoxide.

It is interesting to note that under these conditions and for some amines, even acetophenone was reactive. A possible explanation for the formation of the imine when ketones other than acetone are used relies on a different opening mechanism of the Bargellini epoxide. Indeed, when the steric factor is significant, the epoxide can undergo a C-C cleavage, instead of a C-O one, in order to relief the hindrance (Scheme 21).

**Scheme 22:**
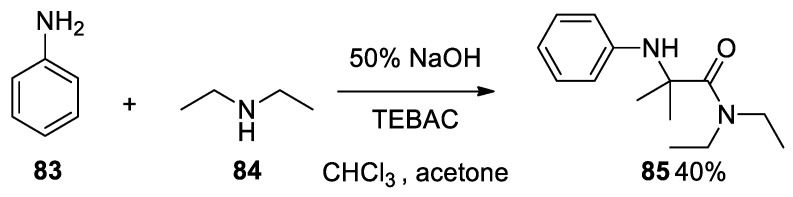
Bargellini reaction using aniline and diethylamine.

Furthermore, it is important to highlight that when aniline **83** and diethylamine **84** were reacted together, contrary to expectations, the less nucleophile aniline attacks first the Bargellini expoxide to form compound **85** [53]. To prove this oddity, we carried out this reaction in our laboratory, confirming the result (Scheme 22).

**Scheme 23:**
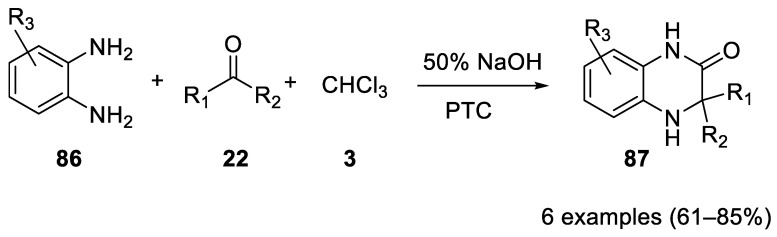
Reaction with *o*-phenylendiamines.

In 1982 *o*-phenylendiamines **86** were reacted in the Bargellini reaction to form 3,3-dialkyl-1,2,3,4-tetrahydro-2-quinoxalinones **87** under PTC (Scheme 23). 

Acetone, cyclopentanone, cyclohexanone and octan-2-one react successfully. Similar results were obtained using trichloromethylcarbinol under mild PTC, allowing a high degree of tolerance towards the substituents R_1_ and R_2_ [54,55].

**Scheme 24:**
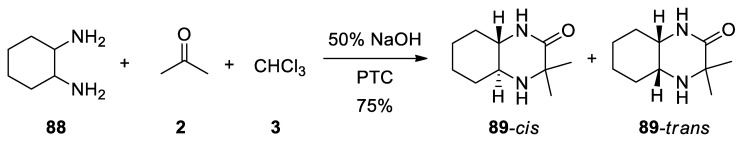
Reaction with 1,2-cyclohexyldiamine.

In the same paper, the reaction of 1,2-cyclohexyldiamine **88** is reported to form the *trans*-3,3-dimethyldecahydro-2-quinoxalinones **89** in 75% yield as a mixture of *cis* and *trans* isomers **89** (Scheme 24).

**Scheme 25:**
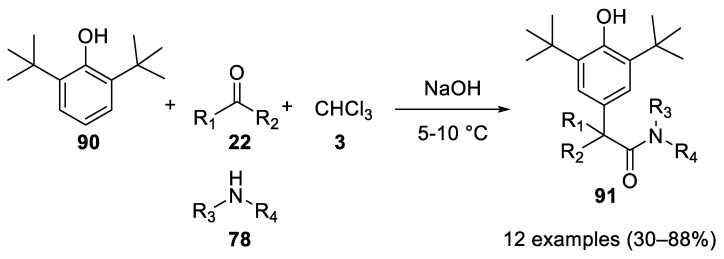
Reaction with 2,6-ditertbutylphenol.

In 2001 strongly hindered phenols, such as 2,6-di-*tert*-butylphenol (**90**) reacted in the Bargellini reaction with the carbon at the *para* position, instead of the sterically hindered hydroxy group. The use of PTC conditions allows for the interception of the acyl chloride with an amine group to give 2,6-ditertbutyl-4-(1,1-dialkyl-1-acetamide)-phenols **91**. Note that the amines do not compete with the phenol in attacking the oxirane intermediate, allowing for a chemoselective reaction [56] (Scheme 25).

**Scheme 26:**
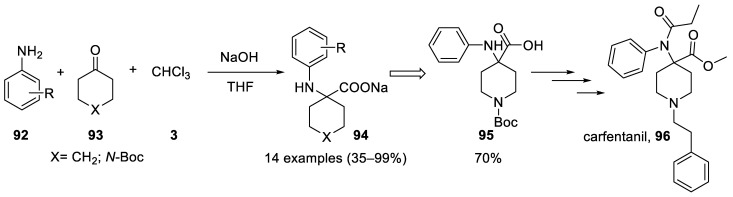
Reaction with anilines and total synthesis of carfentanil using the Bargellini reaction.

Although aniline had been shown to react in the Bargellini reaction, no general reaction with other aromatic amines was reported until 2009, despite the seminal contribution of Banti [40]. Different aromatic and heteroaromatic anilines **92** and cyclohexanone or *N*-Boc-cyclopiperidinone (**93**) was used and shown to react. The sodium salt precipitates in the reaction medium and the work-up consists only in a simple filtration [57] (Scheme 26). Following this approach, an improved synthesis of carfentanil (**96**) has been reported (Scheme 26).

**Scheme 27:**
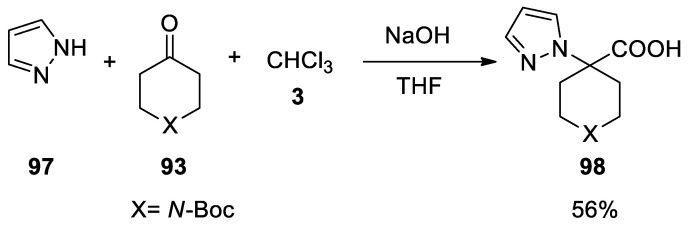
Reaction with pyrazole.

In this paper it was the demonstrated that the poor nucleophilic pyrazole ring **97** reacts under these conditions, affording the Bargellini adduct **98** in good yield (Scheme 27).

A method using KF/alumina instead of sodium hydroxide has been reported. Besides anilines, the reaction performed well with phenols and aromatic thiols [58].

**Scheme 28:**
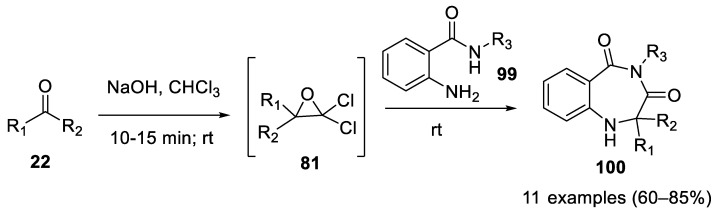
Synthesis of 1,4-benzodiazepine-3,5-diones using Bargellini reaction.

As shown in 2012, 1,4-benzodiazepine-3,5-diones (**100**) can be obtained directly by the Bargellini reaction using 2-amino benzamides **99** as bidentate nucleophile partners [59]. Pre-formation of oxirane followed by the addition of 2-aminobenzamide is mandatory for the success of the reaction. Only acetone and ethylmethylketone were used as carbonyl compounds (Scheme 28).

**Scheme 29:**
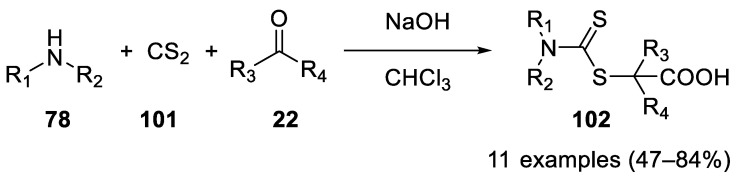
Reaction with dithiocarbamic acids.

In 2012 dithiocarbamic acids, prepared in situ from secondary amines **78** and carbon disulfide (**101**), proved to be valuable nucleophiles in the Bargellini reaction, with acetone, cyclohexanone and cyclopentanone as carbonyl partners [60] (Scheme 29). 

**Scheme 30:**
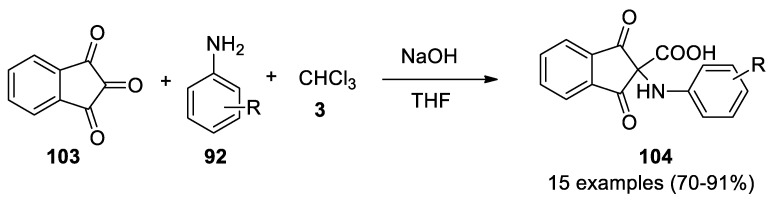
Reaction with ninhydrin.

In 2013 ninhydrin (**103**) proved to be an excellent carbonyl group in the Bargellini reaction with anilines **92** as nucleophiles [61]. The reaction worked with different type of aromatic amines, while with aliphatic ones, such as methyl and ethylamine, yields lower than 20%. Isatin, acenaphthoquinone and 9,10-phenanthrequinone did not react (Scheme 30).

## 4. Applications of the Bargellini Reaction

**Scheme 31:**
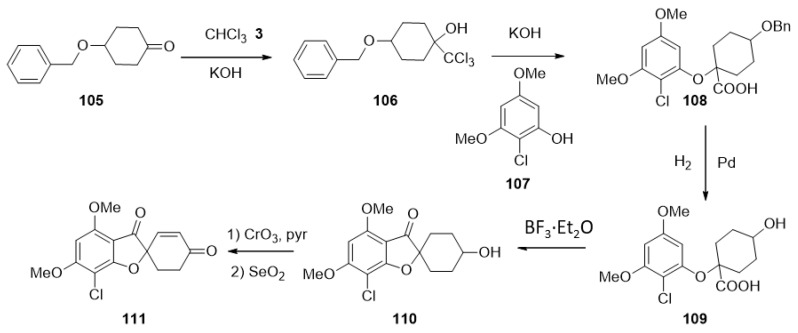
Synthesis of grisandione.

While it is now recognized that the synthesis of griseofulvin was not carried out using the Bargellini reaction, as erroneously stated in several papers, Korner reported the synthesis of grisandione (**111**) and analogues using this transformation [62] (Scheme 31).

**Scheme 32:**
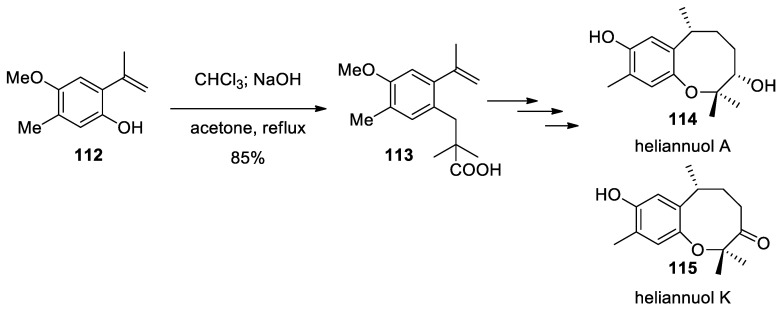
Total synthesis of heliannuols using the Bargellini reaction.

The Bargellini reaction has also been used for the total synthesis of heliannuols A **114**, and K **115** [63,64,65,66,67] (Scheme 32).

**Scheme 33:**
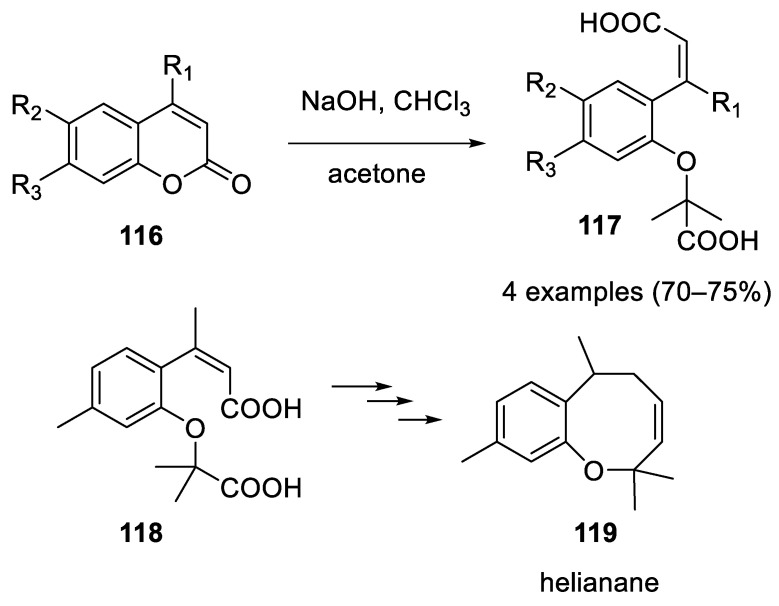
Coumarins can react in the Bargellini reaction.

Interestingly, coumarins **116** can be used as masked phenols in the Bargellini reaction. Basic conditions allow for the lactone opening, followed by reaction with the Bargellini epoxide. By using coumarins there is the advantage of installing an extra acrylic acid functionality. Authors used this novel transformation in the total synthesis of helianane (**119**), a marine sesquiterpene [68] (Scheme 33).

**Scheme 34:**
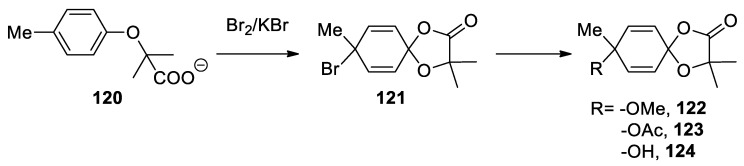
Corey’s elegant synthesis of 2,5-cyclohexadienones.

In 1969, Corey used the product of Bargellini reaction **120** in order to form, under mild reaction conditions compatible with several functional groups, 2,5-cyclohexadienone derivatives **122**–**124**. This reaction is reminiscent of the Birch reduction, but without the need of using sodium and ammonia. In brief, the Bargellini product, as sodium salt, was treated at 0 °C with an aqueous solution of bromine-potassium bromide to give the bromolactone **121**. The latter can be used as intermediate to access other 2,5-cyclohexadienones [69] (Scheme 34). 

**Scheme 35:**
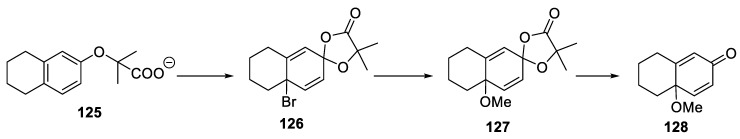
Synthesis of 5,6,7,8-tetrahydro-2-naphtol.

This strategy is exemplified by the elegant synthesis of 4a-methyl-5,6,7,8-tetrahydronaphthalen-2(4aH)-one (**128**), starting from 5,6,7,8-tetrahydro-2-naphtol (**125**) (Scheme 35) [69].

## 5. The Future of Bargellini Reaction

**Scheme 36:**
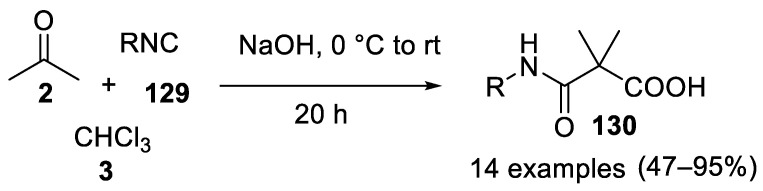
Isocyanides are nucleophilic partners in the Bargellini reaction.

As we have shown in this review, after the discovery of Bargellini reaction with phenols, other nucleophiles proved to be a valuable alternative, further expanding the scope of this reaction. On the other hand, several other nucleophiles have not been reported to react in the Bargellini reaction and could lay undiscovered. Our recent discovery that isocyanides **129** are excellent partners in the Bargellini reaction forming 3-carboxamido-isobutyric acids **130** in a single operation strengthens our belief [70] (Scheme 36).

**Scheme 37:**
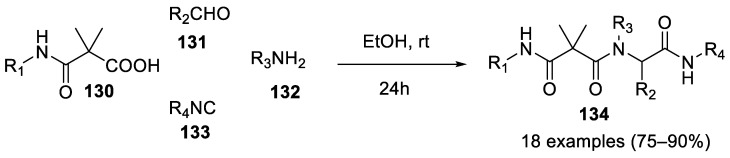
Synthesis of pseudopeptides via a consecutive Bargellini-Ugi reaction.

This reaction has also been recently used in a consecutive Bargellini-Ugi reaction to generate pseudo-peptides [71] (Scheme 37).

In this review, we have shown that different reaction conditions have been developed for performing successful Bargellini reactions, and, capitalizing on our experience on this transformation, we propose a simple scheme to follow when performing Bargellini reactions with unprecedented nucleophiles. Indeed, the attempt to perform this reaction simply using the classical procedure is wrong as it is important to consider the nucleophilic nature of the reactants, the preformation of the Bargellini epoxide, the use of trichloroacetone and the use of PTC conditions. We believe that the reactivity with the Bargellini epoxide has been underestimated due to a not complete survey of the rection conditions. Herein, we propose a set of reaction conditions that need to be used before stating that a nucleophile is not reactive in the Bargellini reaction:

(a) substrate, acetone, CHCl_3_, freshly pulverized NaOH both at 0 °C, room temperature and at reflux;
(b) substrate, acetone, CHCl_3_, freshly pulverized NaOH in THF both at 0 °C, room temperature and at reflux;
(c) substrate, acetone, CHCl_3_, freshly pulverized NaOH 50% in water, dichloromethane, TEBAC under PTC at 0 °C
(d) substrate, acetonechloroform (or related trichloromethylcarbinols), freshly pulverized NaOH, water, at 0 °C, room temperature and at reflux;
(e) substrate, acetonechloroform (or related trichloromethylcarbinols), freshly pulverized NaOH, THF, at 0 °C, room temperature and at reflux;
(f) acetonechloroform (or related trichloromethylcarbinols), freshly pulverized NaOH 50% in water, dichloromethane, TEBAC under PTC at 0 °C.

An advantage of the Bargellini reaction is that this transformation can easily insert a *gem*-dimethyl quaternary carbon. Many natural products contain the *gem*-dimethyl moiety connected with an oxygen atom. This feature seems to be necessary to impart peculiar characteristics to the molecules and a recent review article has highlighted that the *gem*-dimethyl group can be of great help in improving both pharmacokinetic and pharmacodynamic properties [72]. In brief, a *gem*-dimethyl group can reduce the conformational flexibility, and, when in α-position to a carboxylic acid, it can reduce the formation of acyl glucuronides and prevent toxicity problems related to this metabolic pathway.

Finally, union multicomponent reactions (MCRs), which consist of the combination of two or more different types of MCRs in a one-pot process, lead to complex molecules with the concomitant formation of several bonds. To this aim, it is necessary that a functional group compatible with the first transformation be present and inert in the first step, so that it can react in the second reaction, or, alternatively, it is necessary that a functional group be generated in the first step, ready to react in the second transformation. Successful examples of this strategy have appeared in literature, for example the combination of Petasis-Ugi [73,74]. We believe that, due to its peculiar features, the Bargellini reaction has the potential to be used successfully in this strategy as recently demonstrated during the writing of this review article [71].

For all these reasons, we foresee that in the near future the Bargellini transformation will be used more than in the past as an effective means to expand the chemical space accessible by this reaction.
